# ﻿Predator responses to artificial aposematic and cryptic colouration in terrestrial isopods (Isopoda, Oniscidea)

**DOI:** 10.3897/zookeys.1225.121574

**Published:** 2025-02-05

**Authors:** Lenka Skočková, Barbora Ďurajková, Ivan Hadrián Tuf

**Affiliations:** 1 Department of Ecology and Environmental Sciences, Faculty of Science, Palacký University, Olomouc, Czech Republic Palacký University Olomouc Czech Republic

**Keywords:** *Podarcissiculus*, *Porcellioscaber*, terrestrial isopods

## Abstract

Aposematism is a distinctive or warning signal that provides the animal with protection against a potential predator. Aposematic colouration is easier for a predator to remember and to avoid a dangerous and/or unpalatable prey in the future. We investigated whether distinctive colouration has an aposematic function in terrestrial isopods. The common rough woodlice (*Porcellioscaber*) were used as a model species of terrestrial isopods and the Italian wall lizard (*Podarcissiculus*) as a predatory species. To imitate the distinctive colouration on isopods we marked their dorsal plates with yellow dots. The control group of the woodlice were marked with grey spots. Differences in behaviour (observation, manipulation and consummation) and the lizards’ behaviour changes towards aposematically and cryptically coloured prey were analysed. Differences were found in prey observation both between sexes and between prey colours.

## ﻿Introduction

To leave the sea and fully transition to a terrestrial way of life, terrestrial isopods had to develop many adaptations (Hornung 2011). Different ecomorphological types can be distinguished in this group (Schmalfuss 1984). The first type are the “clingers”, which have strong, short pereopods and their body is broad and flat, so they move relatively slowly (genera *Trachelipus*, *Porcellio*). Another type is the “runners”, which, on the other hand, move very fast because they have long pereopods and slender bodies (families Ligiidae, Philosciidae). The species that inhabit the soil belong frequently to the group of “creepers”, they are smaller, have an elongated body and ribs on the dorsal surface. These species need high ambient humidity. The fourth group are the “rollers”, which use conglobation, have a convex body and can roll up (families Armadillidae, Eubelidae). In tropical and subtropical areas, “spiny forms” of isopods are found. They have spikes or thorns on their bodies to protect them from predators and live outside the topsoil (genera *Panningillo*, *Echinodillo*).

Terrestrial isopods have a large number of natural predators. The main predators include centipedes, spiders, various insects, but also insectivorous vertebrates. Predation pressure has helped the isopods to develop various defence and protection mechanisms, whether it be morphological adaptations or behavioural changes (Tuf and Ďurajková 2022). Increased consumption by birds and reptiles is due to an increased need for calcium necessary for eggshell formation (Bureš 1986; Krištín 1992).

### ﻿Colouration of terrestrial isopods

The body colouration of animals has three main purposes; these are thermoregulation, intraspecific communication, and reduction of predation risk. Body colour and colour pattern play a major role in intraspecific communication, e.g., mate recognition or courtship. Colouration is also important in relation to predation; the animal wants to avoid, deter, or confuse the predator (Endler 1978).

Crustacean chromatophores usually contain a variety of pigments. There are different forms of chromatophores (polychromatic, monochromatic, bichromatic) (Knowles and Carlisle 1956). The brown-black pigment in the melanophores of crabs is melanin, but the dark chromatophore pigments in other crustaceans are ommochromes (Fingerman 1965). The pigments in erythrophores and xanthophores are carotenoids, but there are some exceptions; the most common carotenoid in chromatophores is astaxanthin (Fingerman 1965). Pigments may be distributed freely in the exoskeleton or in chromatophores (Chang and Thiel 2015). Chromatophores are often clustered into larger groups, these multicellular clusters are called chromatosomes. Crustacean chromatophores are asymmetric and mononuclear (Chang and Thiel 2015).

The purpose of cryptic colouration in animals is to reduce the possible detection by predators. In many cases, this colouration can indicate that the animal is trying to blend in with its surroundings (Merilaita 1998). Within terrestrial isopods, such colouration is typical for epigeic species with dusk or daytime activity (e.g., *Porcellioscaber*, *Porcellionidespruinosus*, *Trachelipusrathkei*, *Hemilepistusreaumurii*). During the day, cryptic species usually hide in shelters in or on the soil surface (Davies et al. 2012). Some species are capable of colour-change or pattern transformation on the body. Colour-change, which is based on chromatophores (melanophores and leucophores), is triggered by sensory detection from the surrounding environment (Nery and de Lauro Castrucci 1997). The littoral isopod *Ligiaoceanica* uses melanophores to change colour within a circadian rhythm (Hultgren and Mittelstaedt 2015).

In cryptic polymorphism, different colour forms occur within the same species (Veselý et al. 2024). This is because the predator has a certain image in memory (the search image) by which it searches for its prey (Punzalan et al. 2005). Thus, if a predator detects one morph, other individuals with a different pattern are in relative safety (Hughes and Mather 1986; Veselý et al. 2024). This principle protects individuals with rarer colouration, as the predator mainly targets the more numerous colouration pattern of prey. Thus, polymorphism is maintained in the population by predation, as the preference for a particular pattern is determined by the current commonness or rarity of the colouration. However, this principle does not apply to morphs with a high degree of divergence in conspicuousness. Albinos are always conspicuous, even if they are rarer. Within crustaceans, albinism is a relatively rare phenomenon (Geiser 1932). In the isopods, this phenomenon is common in species that live in caves or deeper in the soil. In some species, albinos are also rarely found in natural conditions, but they are regulated by a higher level of predation (Achauri 2009).

Aposematism is a conspicuous warning colouration or other type of warning signal by which an individual alerts potential predator to its (real or perceived) inedibility or toxicity. There are colour combinations that are typical: black or dark brown in combination with yellow, red or orange, or sometimes even white. Stripes or spots on the bodies of aposematically coloured individuals are also common (Davies et al. 2012). Aposematic colouration uses colour contrast, where we can observe as differences in shades or saturations of colour between a given organism and the environment in which it lives. It can also make use of luminance contrast, where the amount of light reflected from the organism and its surroundings varies. Colour contrast is considered important for the effectiveness of aposematism, especially in the case of avian predators and diurnal lizards (e.g., Lacertidae), which have tetrachromatic vision. Conversely, for colour-blind predators, luminance contrast is an important factor that enables them to detect aposematic prey (Prudic et al. 2007). Aposematic colouration is very conspicuous to predators, they can easily spot and recognise it, but also remember it very easily (Prudic et al. 2007). Predators avoid such distinctive individuals, either through innate neophobia (individual avoids things it does not already know) or learned avoidance of a particular pattern or colour (Vickers et al. 2021).

The light spots on the bodies of woodlice are typical for some species. They are often found on species that are active on vertical surfaces, such as rocks or tree trunks, and are typical of the morphotype “clingers” (*Oniscusasellus*, *Porcelliospinicornis*), but also “rollers” (*Armadillidiumpictum*, *A.opacum*). Vividly coloured are also the vegetation-dwelling “spiny forms” (e.g. *Pseudolaureolaatlantica*).

One theory of the origin of aposematic colouration is that the cryptically coloured toxic species was accidentally consumed by predators who mistook it for a harmless species. They preferentially avoided the more conspicuous specimens that they could readily identify. Through this selection, the conspicuous pattern gradually dominated the prey population (Davies et al. 2012). This theory appears to be applicable to Mediterranean terrestrial isopods, where a similar colour pattern also occurs in unrelated species. The shared colour pattern in syntopic species of millipede, pillbug, and spider was pointed out by Levi (1965) as Müllerian mimicry. If several species have the same predators, therefore the more similar species there are, the more likely the predator will learn to recognize them and not attack them (Davies et al. 2012).

Evidence that distinctive colouration has an aposematic function in terrestrial isopods is still lacking (Tuf and Ďurajková 2022). Yellow and white patches are relatively common in the genera *Armadillidium* and *Porcellio*, but a pattern of a combination of black (dark purple-brown) and white has been observed in related species in West Africa (Schmalfuss and Ferrara 1982) too.

### ﻿Cognitive abilities of vertebrate predators

The cognition encompasses a set of mental processes that include perception, learning, long-term memory, working memory, attention and, last but not least, decision making (Dukas 2004; Shettleworth 2010). By learning and then remembering aposematic prey, predators can avoid it in the future (Shettleworth 2001).

Birds and diurnal lizards are primarily visual creatures. Both groups have tetrachromatic vision, with four types of cones (Chen and Goldsmith 1986; Pérez i de Lanuza and Font 2016). Taste receptors are relatively poorly developed in birds, with taste buds located at the root of the tongue, in the posterior palate, and in the pharyngeal mucosa (Bill 2007). Olfaction in reptiles is provided by the vomeronasal organ (via the forked tongue) and the olfactory mucosa in the paired nasal cavity (Vitt and Caldwell 2014).

The main aim of this work is to find out whether the colour of the woodlice has an aposematic meaning. We wanted to find out how reptilian predators would react to presented prey and whether colouration would play a role in prey selection. We also investigated whether predatory behaviour and foraging motivation would change in a model predator species over the course of the experiment. We tested the following hypotheses: 1) there is difference in lizards’ behaviour towards aposematic and cryptic prey, 2) isopods with aposematic colouration are less consumed than isopods with cryptic colouration, 3) there is no difference in prey consumption between males and females of lizards, and 4) predatory behaviour and foraging motivation of lizards can change throughout the experiment.

## ﻿Material and methods

### ﻿Model species

The model prey species was the common rough woodlouse (*Porcellioscaber*). This species has a cosmopolitan distribution (Capinera 2001). It is coloured in shades of brown and grey but may show small patches of colour (black, red, orange, yellow) (Capinera 2001). A number of colour forms have been bred in hobby breeding. Birds, lizards, newts, spiders, beetles, centipedes, and shrews are considered to be the main predators of woodlice.

The collection of woodlice took place in the autumn of 2022 in Olomouc. They were subsequently kept in plastic boxes with lids, inside there was soil, leaves, and shelters (bark, stones), the substrate was kept moist in places, with a constant temperature (18–22 °C). Individuals of 8–10 mm in length were used in the experiments to make them attractive to the predators of interest.

The model reptile predator species was the Italian wall lizard (*Podarcissiculus*). Adults can reach lengths of up to 25 cm and weights of 15 g. The original distribution area is thought to be the Apennine Peninsula (Rivera et al. 2011), but it is now also widespread in the Iberian Peninsula and North Africa. This species is popular among breeders, and the lizards are fed mainly by mealworms and crickets.

Experiments were conducted with four young immature lizards (2 males and 2 females). These lizards were naïve and kept in captivity from their birth. Animals were housed in a terrarium (120 cm × 50 cm) with an 8:16 light regime at the time of the experiment, and the ambient room temperature, but the terrarium also contained a heating pad. Inside the terrarium was a sandy lignocel mixture as a substrate, a water bowl, and bark pieces as a shelter. Before the start of the experiment, the animals were fed by crickets. During the experiment, the lizards were fed only during individual trials to see if their feeding behaviour would change.

### ﻿Experiment

**Figure 1.**
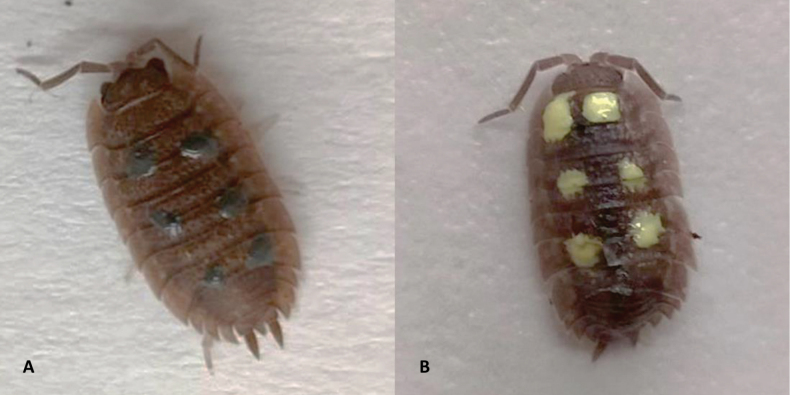
Individuals of common rough woodlouse (*Porcellioscaber*) grey painted as cryptic (**A**) and yellow painted as aposematic prey (**B**).

The experiments were conducted from 24 October to 30 November 2022 according to standard procedures (Exnerová et al. 2006; Veselý et al. 2006; Dolenská et al. 2009). Prior to the experiment, predators had to be placed in empty transparent plastic boxes (57 × 39 × 28 cm) at least two hours before the experiment to ensure habituation and that the experiment was not influenced by fear of the unfamiliar environment (Exnerová et al. 2006). The predators had access to water throughout the two hours. Furthermore, it was necessary to paint the woodlice. Six yellow dots were made on the dorsal plates with nail polish (reminiscent of the colouration of *Porcelliohaasi*). The control group consisted of isopods with the same number of spots, painted with grey nail polish (Fig. 1). Thus, both groups were equally altered and differed only in vivid (aposematic) and cryptic colouration. After drawing dots on the isopods, the polish was allowed to dry for at least an hour so that its odour would not interfere with the experiment. As polish nail can affect activity of isopods for more than week (Drahokoupilová and Tuf 2012), fresh new isopods were dotted before each experiment. Its activity one hour after dotting resembled that one of unpainted isopods.

We used selection tests to test whether the isopods’ vivid colour has an aposematic function. The selection tests consisted of releasing five aposematically (grey–yellow experimental group) simultaneously with five cryptically (grey–grey control group) painted isopods into the box where one predator was placed, and then observing and recording the predator’s behaviour. Before the insertion of the painted prey, the predator was presented with a mealworm to control feeding motivation. In one experiment each lizard experienced a series of ten tests in the following order: mealworm – painted (grey and yellow) isopods – mealworm – painted isopods, etc. Unpainted prey (*P.scaber* without drawn dots) was also presented to predators, always at the beginning and end of the experiment. One test lasted for seven minutes. Each predator was tested in this way twice a week for five weeks. Altogether 10 experiments consisting of 10 tests were performed with each lizard giving a total of four hundred observations for each behaviour. Experiments were done during day hours in laboratory with ambient light and temperature. All four lizards were tested on the same days with a time interval of 1.5 hours. Entire experiments were recorded on camera (Niceboy VEGA X PRO) for control possibility.

Predator behaviour was divided into three categories: 1) prey consummation, which involved the direct eating of prey 2) prey manipulation, which involved biting the isopod or devouring it and then spitting it out, and 3) prey observation, which involved turning the head to follow the prey or chasing the isopod. Unless consummation was preceded by a prolonged examination of the prey, this behaviour was not considered as observation. The trials with mealworms were not analysed, they were just used to control for foraging motivation during the whole session.

### ﻿Data analysis

Graphical representations of behavioural changes (consummation, manipulation, observation) in predators over the course of the experiment were made using MS Excel.

The aim of the two-factor analysis of variance (ANOVA) was to determine whether there were differences between the behavioural factors (consummation and observation). The significance level for the analysis of variance was set at 5%. Predator sex (♂, ♀) and prey colour (A, C) were designated as factors. A separate ANOVA was conducted for each behavioural type. A normality check was performed using graphical display and Levene’s test to verify that the assumptions of the ANOVA were met. Due to the failure of the data to follow a normal distribution for one type of behaviour (manipulation), a Kruskal–Wallis test (non-parametric one-factor ANOVA) was performed. The Kruskal–Wallis test tests one factor at a time and compares whether there are differences in variances between the selected groups. The factors tested were predator sex (M, F) and prey colour (A-aposematic, C-cryptic). Two such tests were performed on each factor, and the significance level was set at 5%.

Tukey’s test (multiple comparisons test) was performed to determine which factors (and combinations of factors) may influence predator behaviour change. R-Studio software was used to analyses of variance and Kruskal–Wallis tests, followed by graphical representations.

## ﻿Results

We presented the naïve lizards with yellow painted (aposematic colouring), grey painted (cryptic colouring) as well as with unpainted (*P.scaber* without drawn markings) isopods. During the observed period (i.e., five weeks), the consumption rate of unpainted prey decreased from 65% to 5%.

**Figure 2.**
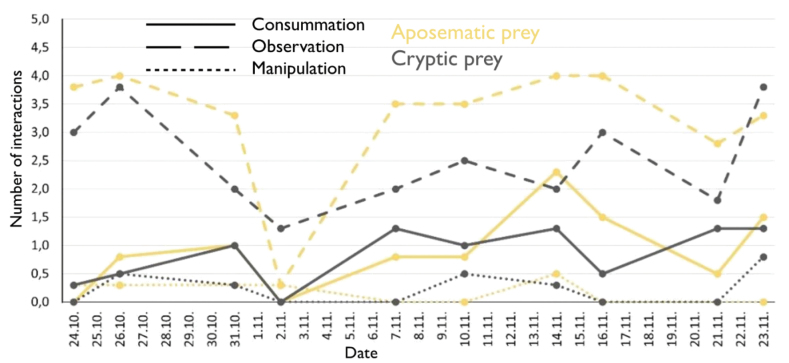
Average number of measured interactions of all tested predatory behaviours of Italian wall lizards (*Podarcissiculus*) towards aposematically or cryptically coloured common rough woodlice (*Porcellioscaber*).

The most frequent type of lizards’ behaviour was the observation of prey regardless of its colouration (Fig. 2), while manipulation of the presented isopods was the least frequent. Consumption of cryptic prey was higher if it followed a longer time interval (four or more days between experiments) (Fig. 2). For aposematic prey, this relationship was not as apparent. Due to an injury to one female, two of her last trials (24.11. and 30.11.) were not included in the behavioural display.

### ﻿Differences in lizard behaviour

Two hundred of tests for both males and females (two individuals of each sex, 10 experiments of 10 tests) presented to both types of prey simultaneously were compared. Normality of the data was not confirmed by graphical display, which is not a major obstacle in ANOVA. The analysis of variance is robust to a small failure to meet this assumption, especially if the samples have a size of at least 20, which samples met with exception of data about prey manipulations.

**Figure 3.**
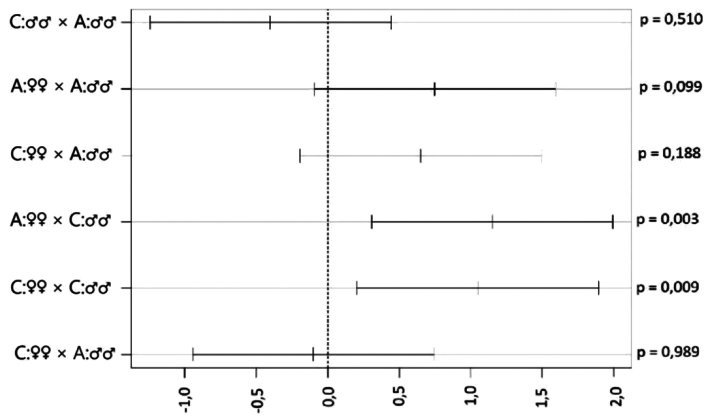
Visualisation Tukey’s post hoc tests of consummation of painted common rough woodlice (*Porcellioscaber*) by males and females of Italian wall lizard (*Podarcissiculus*). The mean difference between means of both categories with 95% CI and p-values are presented. Treatment of prey: A – aposematic, C – cryptic.

There were statistically significant differences between predator sexes in consummation (ANOVA, *F* = 15.72, *p* < 0.001) but no differences between aposematic and cryptic prey. Significant differences in behaviour are only within predator sex (Fig. 3). Females consumed more prey, both cryptic (significantly) and aposematic (unsignificantly).

The manipulation was relatively rare category thus frequency was tested by the Kruskal–Wallis test, nevertheless, there were no differences between males and females manipulating prey, as well as between aposematic and cryptic prey manipulated (*p* > 0.05).

**Figure 4.**
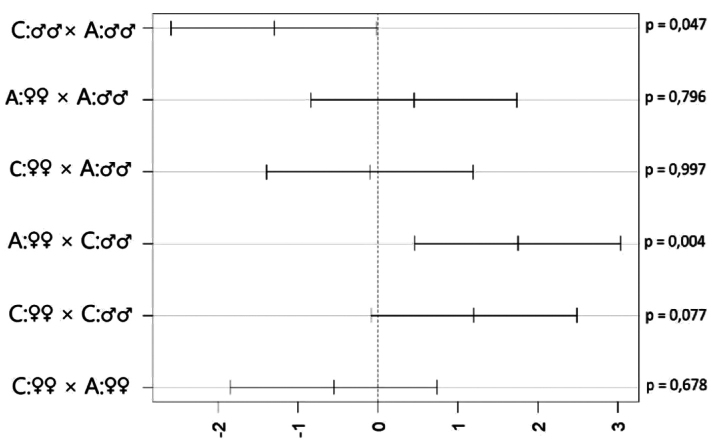
Visualisation Tukey’s post hoc tests of observation of painted common rough woodlice (*Porcellioscaber*) by males and females of Italian wall lizard (*Podarcissiculus*). The mean difference between means of both categories with 95% CI and p-values are presented. Treatment of prey: A – aposematic, C – cryptic.

On the other hand, observation of prey differs significantly between male and female lizards (ANOVA, *F* = 5.65, *p* = 0.020), when females were more interested in observing potential prey as well as between cryptic and aposematic prey (*F* = 7.10, *p* = 0.009), when aposematic prey was more focused. Males observed less cryptic prey than aposematic prey (Fig. 4, *p* = 0.047). Furthermore, there was also a significant difference between females more observed aposematic prey than males observed cryptically coloured prey (*p* = 0.004).

## ﻿Discussion

We tested the effect of prey colouration on predator behaviour by using choice tests in which lizards were presented with yellow painted woodlice to determine whether it would have an aposematic function.

Already during the interaction with the unpainted prey, it was evident that the predators found the isopods unpalatable; the lizards spat out the prey and chewed it for a long time. This behaviour was also observed in the anole (*Anoliscarolinensis*) after consuming the bugs *Neacoryphusbicrucis* and *Lopideainstabilis* (McLain 1984). Woodlice are themselves disgusting, as they produce ammonia, which they excrete from the body via vapours (Sutton 1972) and also a smelly sticky secretion from repugnatory glands. Many animals that exhibit aposematic signals also aid in deterring predators with chemical compounds such as alkaloids (Holloway et al. 1991) or cardenolides (Reichstein et al. 1968). When presented with unpainted prey at the beginning of an experiment, predators consumed more of it than at the end of the experiment. This could have been due to being hungry or curious at the beginning, they certainly did not show the neophobia typical of avian predators (Veselý et al. 2024). In contrast, by the end of the experiment, they had experienced large amounts of presented isopods as distateful prey, and although their foraging motivation (tested on mealworms) was often proven, they were mostly no longer willing to consume the isopods. In experiments with reptilian predators, they orient themselves not only by sight but also by smell, so they may perceive different aposematic signals than birds (Reznick et al. 1981; Hasegawa and Taniguchi 1994; Bonacci et al. 2008; Lee et al. 2018).

For all lizard individuals, the most common behavioural type was observation, with aposematically coloured and also cryptically coloured prey. Less frequently, manipulation or direct consummation of isopod occurred. Prey must move to get their attention, hence the high number of sightings (Sexton 1964). During actual consumption, the odour of the prey plays a large role. Prey odour may be one of the components of aposematic signals in some species (Tseng et al. 2014).

Throughout the experiment, there was no increase in food motivation with the intensity we expected. McLain (1984) conducted experiments with anoles (*Anoliscarolinensis*) that he starved for three days before each subexperiment to induce increased appetite. In this condition, the lizards could disregard the odour of prey and attend more to its colouration. Although the rate of interaction with prey increased slightly over the course of the experiments, we did not observe any major changes in their behaviour. On average, males showed less interest in prey than females, which may be due to the different metabolic energy requirements of males and females. Namely, the size of females during the breeding season may affect the number of clutches per year (Galan 1997).

One female lizard had a hind limb injury, so she missed two partial trials (31.10. and 2.11.). At that time the female was fed mealworms to give her enough energy to recover. In subsequent trials she showed a marked increase in interest in the presented isopods, which was probably due to the increased energy required for full recovery.

In experiments with Taiwan japalure (*Diplodermaswinhonis*), researchers found that there was a difference in prey generalization between males and females (Ko et al. 2020). The individuals used in the experiments were from the wild. As part of the experiments, they were presented with crickets that were dyed red and green, and were infused with chemicals that ensured that the prey was unpalatable. They were also presented with control prey (black). The females were more cautious after exposure to the toxic prey, and avoided the control group of crickets as well. Males, on the other hand, took more risks and tried the prey even assuming that it might be unpalatable. The researchers explain these differences in behaviour between males and females by the fact that males living in the wild have to defend their territory, which is energetically demanding for them; they are also restricted to hunting only in their territory, so they have to take more risks when choosing food because of limited resources. Females may move across territories of different males, so may be more conservative in their food choices (Ko et al. 2020). In contrast, we observed greater activity by females within as well as outside of sub-trials. Females observed and consumed presented prey to a greater extent than males. The feeding motivation of our female lizards seemed to be more influenced by their young age. These young males had yet to defend any territory. Although the difference in the observation of cryptic and aposematic prey was significant, it was significant only for males.

The predator’s size influences feeding behaviour too. Exnerová et al. (2003) found in experiments with *Pyrhocorisapterus* bugs that smaller insectivorous birds (*Parusmajor*, *Cyanistescaeruleus*, *Erithacusrubecula*, *Sylviaatricapilla*) discriminate well between aposematically and cryptically coloured prey, in contrast to larger (*Turdusmerula*) or granivorous birds (*Fringillacoelebs*, *Chlorischloris*, *Emberizacitrinella*). The smaller birds showed nausea when consuming large quantities of unpalatable prey. Perhaps even lizards, after reaching a larger size, will stop being picky and will consume or even discriminate more readily.

In conclusion, the results show that there are differences in prey consumption between the sexes of lizards. Differences were also found in prey observation both between sexes and between prey colour.

In future experiments on this topic, it would be useful to conduct more experiments with different sized lizards. It would also be useful to combine painted prey and preferred prey, such as cockroach nymphs, in selection tests. It would be appropriate to use a different type of prey colouring, such as mixtures of tapioca starch and food colouring, which are free of distinctive taste and odour (Ko et al. 2020).

## ﻿Additional information

### Author ORCIDs

Ivan Hadrián Tuf https://orcid.org/0000-0003-0250-0482

Barbora Ďurajková https://orcid.org/0009-0004-3269-7540

### Data availability

All of the data that support the findings of this study are available in the main text.
